# The Designation Degree of Tool Wear after Machining of the Surface Layer of Duplex Stainless Steel

**DOI:** 10.3390/ma14216425

**Published:** 2021-10-26

**Authors:** Tomasz Dyl

**Affiliations:** Department of Marine Maintenance, Faculty of Marine Engineering, Gdynia Maritime University, Morska Street 81-87, 81-225 Gdynia, Poland; t.dyl@wm.umg.edu.pl

**Keywords:** surface layer, machining parameters, wear of a cutting tool, duplex stainless steel, DSS

## Abstract

This paper presents problems related to the machining of the surface layer. It is important that steel structures are used in material engineering and machine construction. Austenitic, ferritic, martensitic and two-phase corrosion resistant steel was widely used in the petrochemical and shipbuilding industries. Duplex stainless steel was used in sea water and acid installations. The shafts of centrifugal pumps can be made of stainless steel and are used in acid or seawater pumps. The surface layer of corrosion resistant steel shafts must have a low surface roughness. Machine elements made of stainless steel, and in particular of the duplex type, are difficult-to-cut materials. This article aims to study the effect of parameters on tool life and tool wear. The influence of technological parameters such as depth of cut, cutting speed, feed on maximum value tool wear is presented. The treatment of the surface layer was performed using carbide inserts. The highest wear and the lowest roughness were used as selection criteria. This problem is a significant issue due to the ability of the machine parts for the required reliable operation of devices and machines. To determine the degree of tool wear to increase of the surface layer quality after shaping DSS.

## 1. Introduction

This article presents issues related to the processing of the surface layer. Stainless steel structures can be used in materials and mechanical engineering. The ferritic, austenitic, martensitic and two-phase corrosion resistant steels are highly used in the metallurgical, petrochemical, machine-building and shipbuilding. Two-phase stainless steel is used in acid and sea water installations. The shafts of centrifugal pumps can be made of stainless steel and are used in acid or sea water pumps. It is important that the surface of the stainless steel shafts has a very low surface roughness. Machine parts made of stainless steel, in particular of two-phase duplex stainless steel, are a material that is difficult to cut. The article analyzes the effect of technological parameters on tool life and tool wear. The effect of the parameters: depth of cut, feed, cutting speed on the tool wear of the flank surface were determined. The treatment of the surface layer was performed using cemented carbide inserts with a titanium nitride coating. The lowest roughness and the highest wear is used a selected criterion. This problem is significant for the ability of the components to perform the required reliable operation of the machine parts and to determine the degree of tool wear to increase the surface layer quality after machining duplex stainless steel. The duplex stainless steels one of the rapidly progress groups of this materials that they are characterized by the combination of high strength and high corrosion resistance and comparatively small cost.

The chromium-nickel alloys (duplex stainless steel—DSS) are in the proportion of the components that have an equilibrium of the volume fractions of austenite and ferrite [1,2,3]. DSS to be difficult-to-cut materials. The publications [4,5,6] investigate the surface integrity features of duplex stainless steel after turning tool life and wear and workpiece surface roughness was presented. These works described, in great detail, blade wear for duplex stainless steel. Determination of tool wear is presented in a lot of works [7,8,9,10,11]. The analysis of issues related to the new two-phase stainless steels is described in detail in works [12,13]. The influence of the morphology and crystallization mechanism of the sigma phase on properties of DSS and cast duplex stainless steel was determined. The tool wear after turning follows on the lot of faces with direct contact with the workpiece which causes the decrement of the tool surface. Issues of tool wear and surface topography after machining are described in many publications [14,15,16,17,18,19,20,21,22,23]. In papers [24,25,26,27] evaluation inserts after turning duplex stainless steel and the effect of flood and cutting fluid MQL were presented. In this work, experimental investigation of machining parameters used in turning duplex stainless steel was performed. Therefore, defining the tool wear was very important. However, the analyzed article presents other solutions for cutting tools. This essentially introduces new considerations to solve the problem of machining duplex stainless steel. It can be assumed that this is an important issue when designing technological processes for cutting difficult-to-cut steel. The determined are indicators of tool wear cutting edge after turning. The distance between side cutting edge and the maximum distant-rake face wear (KB) and flank wear (VB) was presented.

## 2. Experimental Materials and Methods

The experimental research in the laboratory of engineering production was carried out. The cylindrical surfaces were prepared on CDS 500 × 1000 universal lathe. Technological parameters based on the references and our own studies were designated. Two-phase steel has the chemical constitution and resistance to corrosion similar to austenitic and the physical properties similar to those of ferritic. The investigations were made for samples from X2CrNiMoCuN 25–7–4 (AISI 2507), with the chemical constitution and mechanical properties given in Table 1.

Determining the appropriate surface roughness was dependent on the geometry of the cutting edge, the type of tool and the material to be processed. The samples with DSS after turning are generally short-lived of cutting inserts. Therefore, it was important to define the length of spiral cutting. Length of spiral cutting can be calculated from the formula:

(1) $$LSC=\frac{\Pi \;D_{m}l_{m}}{f}\;\frac{1}{1000}$$

where:

*LSC—*length of spiral cutting, (m); (*LSC* = 9.75 m);

*D_m_*—the diameter of the workpiece, (mm);

*l_m_*—the length of the cutting materials, (mm);

*f*—the feed rate (mm/rev).

The condition for choosing the correct cutting inserts’ geometry, shape and type for machining was the surface roughness profile. The limit value should be complied with (from *Ra* = 0.16 μm to *Ra* = 1.25 μm) and extreme conditions tool flank wear (VB = 0.2 mm). That types of inserts were proposed for finishing machining: CCMT09T308-MM, CCMT09T308-UM, CCMT09T304-UM (Table 2), where all are made of sintered carbides 2025 grade a CVD coting Ti(C,N)/Al_2_O_3_/TiN (2 μm/1.5 μm/2 μm). The tools are characterized by parameters: nose radius r_ε_ = 0.4 mm–0.8 mm; tool cutting edge angle *κ_r_* = 90°; tool included angle *ε_r_* = 80°; rake angle *γ* = 6°–7°; flank angle *α* = 7°. Machining parameters were designated based on references and our own research on machining: depth of cut *a_p_* = 0.5 mm; feed rate *f* = 0.1 mm/rev–0.2 mm/rev; cutting speed *v_c_* = 50 m/min–100 m/min, on the extreme tool wear flank VB. The insert type CCMT where all of sintered carbides 2025 grade were carried out.

The parameters of surface roughness by HommelTesterT1000 profilometer (SEMPRE GROUP Ltd., Gloucester, UK) were measured. The parameters measurements to the principles comprised in ISO standards were performed.

Measurements and observations of the turning samples tool wear using the Scanning Electron Microscopy (SEM) Zeiss EVO MA 15 (ZEISS, Oberkochen, Germany) were carried out.

The microscope is intended for metallographic and structural studies of solids. The instrument allows the imaging metallographic specimen with a resolution of 3 nm at 30 kV. Magnification was possible from five to one million times. This microscope enables the observation of the metallographic specimen with a mass up to 500 g (in all directions and at full mobility of the table) or up to 5 kg (is limited the table move only to the XY axis directions). The microscope enables observation of metallographic specimen as well as measurements of quantities geometric. This device was furthermore equipped with an Energy Dispersive Spectrometer analyzer. EDS XFlash 6/30 by Bruker (BRUKER Corporation, Billerica, MA, USA) outspreads the research possibilities of SEM with accurate and chemical constitution of the observed surface metallographic specimen.

**Figure 1:**
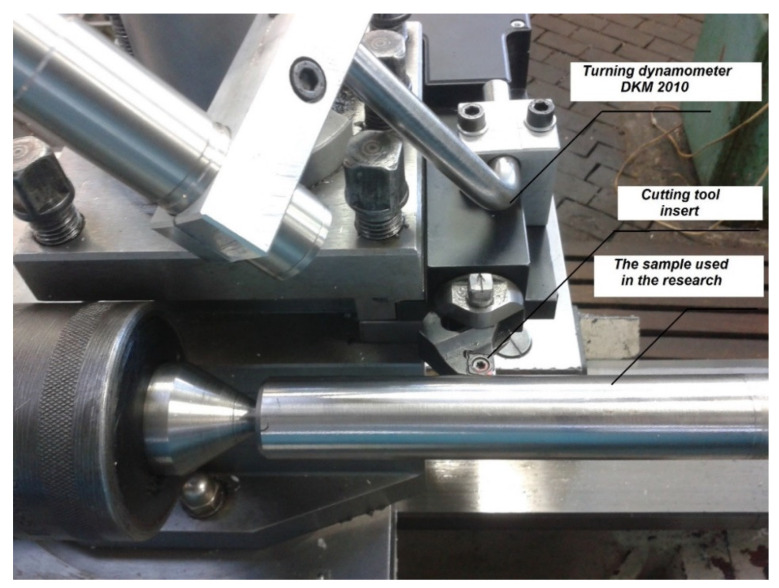
The turning dynamometer DKM 2010 of the outside cylinder surfaces samples in the research and cutting tool insert.

The research laboratory stand for turning of outside cylindrical surfaces of DSS is presented in Figure 1. Longitudinal turning is a machining that includes shaping the surface properties of using tools with the right cutting edge geometry. The indispensable characteristic of longitudinal turning is the linear move of the tool and the matching of the rotational of the workpiece. The surface is appropriate shape and dimensions in the used cutting conditions after machining.

The finishing used to form the surface layer of the parts machines is machining. A tool with the right geometry cutting edge is used. The turning includes shaping of surface properties. The monometallic tool or bimetallic tool or with cutting inserts is used. The surface layer from the workpiece was formed. The important aspect of machining is the correct relationship of linear movement of the tool and rotation of the workpiece. Force was measured using tool dynamometer turning DKM 2010 (TeLC, Technical eLectronic Constructions, Unna, Germany). The cutting tool’s force measured up to 2 kN and temperatures on the tool’s cutting edge ranged from 300–800 °C.

## 3. Results of Experimental Investigation

The turning of duplex stainless steel that the machining parameters impact of the surface quality and tool wear was determined. After the testing carried out, with the assumed processing parameters of the finishing of shaft with DSS. It was determined that the insert type shape, type and grade of the tools as well as the technological parameters have an influence on the reduction of roughness and the correct distribution of cutting forces and tool wear of the cutting edge. Many roughness parameters were defined. The results of measurements of surface roughness of the samples from DSS after turning (for values of feed rate and cutting speed and the constant cutting depth *a_p_* = 0.5 mm) are presented in Table 3. Parameter of surface roughness *Ra* is the arithmetic mean roughness value from the amounts of all profile values. Parameter of surface roughness *Rz* is maximum height of profile average value of the five measurements.

**Figure 2:**
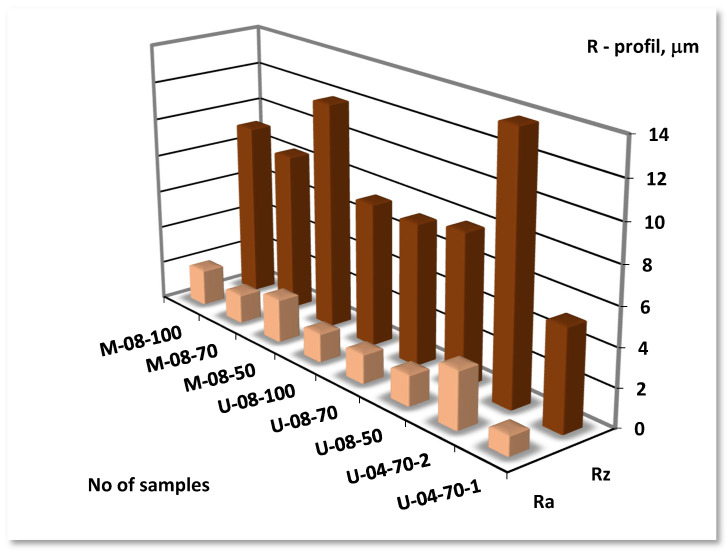
*Rz*—maximum height of profile; *Ra*—arithmetical mean deviation of the roughness profile for samples DSS after turning.

After machining with cutting insert type CCMT09T308-UM 2025 (CC2) for the technological parameters: depth of cut *a_p_* = 0.5 mm; cutting speed *v_c_* = 70 m/min; feed rate *f* = 0.2 mm/rev; lowest values *Ra* (parameter arithmetical mean deviation of the roughness profile—*Ra* = 1.55 μm) were determined. After machining with the technological parameters: depth of cut—*a_p_* = 0.5 mm; cutting speed—*v_c_* = 70 m/min; feed rate—*f* = 0.1 mm/rev; using a CCMT09T304-UM (CC3) of a slight values of the parameter (*Ra* = 1.05 μm) were determined. Cutting insert carbides of the 2025 grade with CVD coatings (Ti(C,N)/Al_2_O_3_/TiN) was used. Turning inserts by the following features: rake angle *γ* = 6°, *γ* = 7°; flank angle *α*= 7°; nose radius *r_ε_* = 0.4 mm, *r_ε_* = 0.8 mm; cutting edge angle *κ_r_* = 90°; tool included angle *ε_r_* = 80°, were characterized. This turning insert tool, the smallest flank wear VB = 0.036 mm and rake face wear KB = 0.018 mm was received. Surface roughness reduction was equal to about three times. It is the extreme value among the show range of studies. *Ra—*arithmetical mean deviation of the roughness profile and *Rz*—maximum height of profile on Figure 2 was presented.

**Figure 3:**
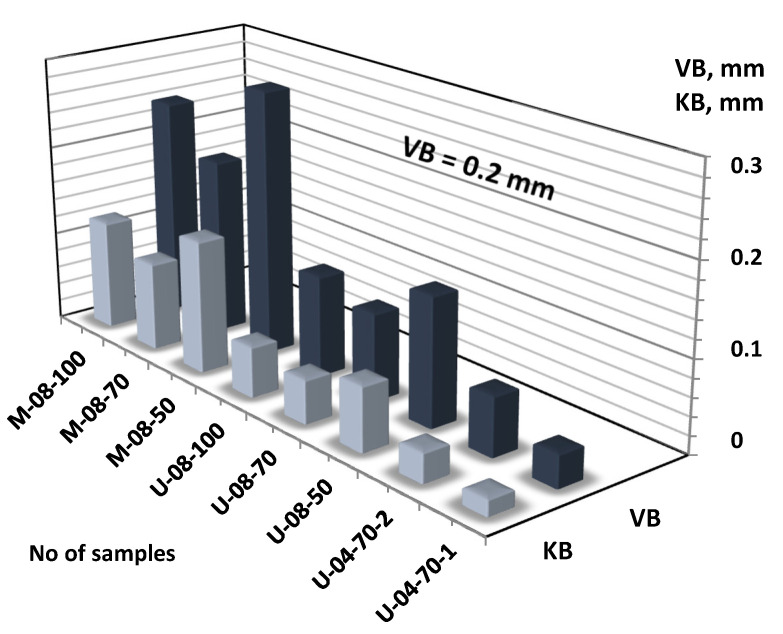
VB—flank wear and KB—rake face wear for samples DSS after turning.

The minimum roughness and the maximum wear was recommended. The surface roughness profile was the criterion for choosing turning insert types for finish, which should be within the range from *Ra* = 0.16 μm to *Ra* = 1.25 μm. The second criterion was of the extreme flank wear VB = 0.2 mm. Therefore, the tools used for experiment testing that meet the wear condition can be concluded. Turning tools met the conditions with the exception of No insert CC1 (No samples: M-08-100 and M-08-50) (Figure 3). To reduce surface roughness after machining, a cutting speed of *v_c_* = 70 m/min for feed rate *f* = 0.1 mm/rev and depth of cut *a_p_* = 0.5 mm for insert type CCMT09T304-UM 2025 (CC3) where is the smallest flank wear VB = 0.036 mm should be used.

**Figure 4:**
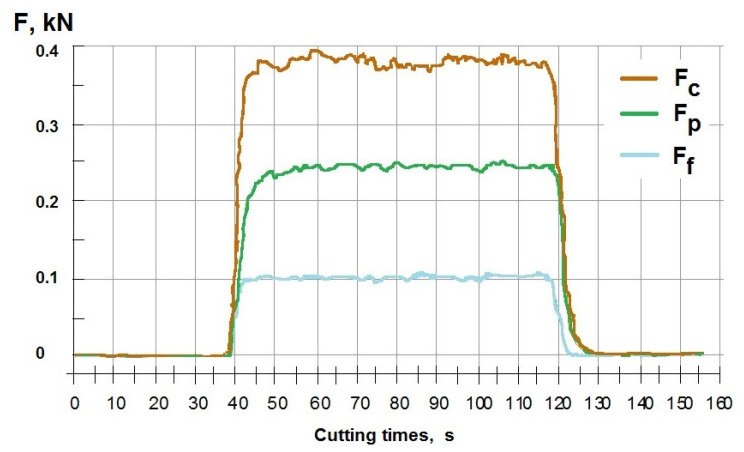
Cutting forces after turning for insert CC3 for the samples with DSS, for *f* = 0.1 mm/rev, *a_p_* = 0.5 mm; *v_c_* = 70 m/min (*Ra* = 1.05 μm).

Figure 4 presents examples of cutting forces for insert number CC3 with machining of DSS samples, for technological parameters: depth of cut *a_p_* = 0.5 mm; feed rate *f* = 0.1 mm/rev; cutting speed *v_c_* = 70 m/min. Cutting force shoulders maximum values of F_c_ = 0.39 kN, when the feed force has the minimum value of F_f_ = 0.1 kN, and the resisting force F_p_ = 0.25 kN. It is characteristic of the study program type of distribution of cutting forces. Machining forces in cutting was carried out using dynamometer DKM2010.

The experimental study has determined that using a CCMT09T304-UM (CC3) and parameters (*a_p_* = 0.5 mm; *f* = 0.1 mm/rev; *v_c_* = 70 m/min) results in the reduction of cutting forces and surface roughness, tool wear of the cutting edge, advantageously influences the chip shape and breakability, and extends the cutting tool life. For proposed parameters technological wasn’t influence of increased chip breaking when machining by turning occurs.

**Figure 5:**
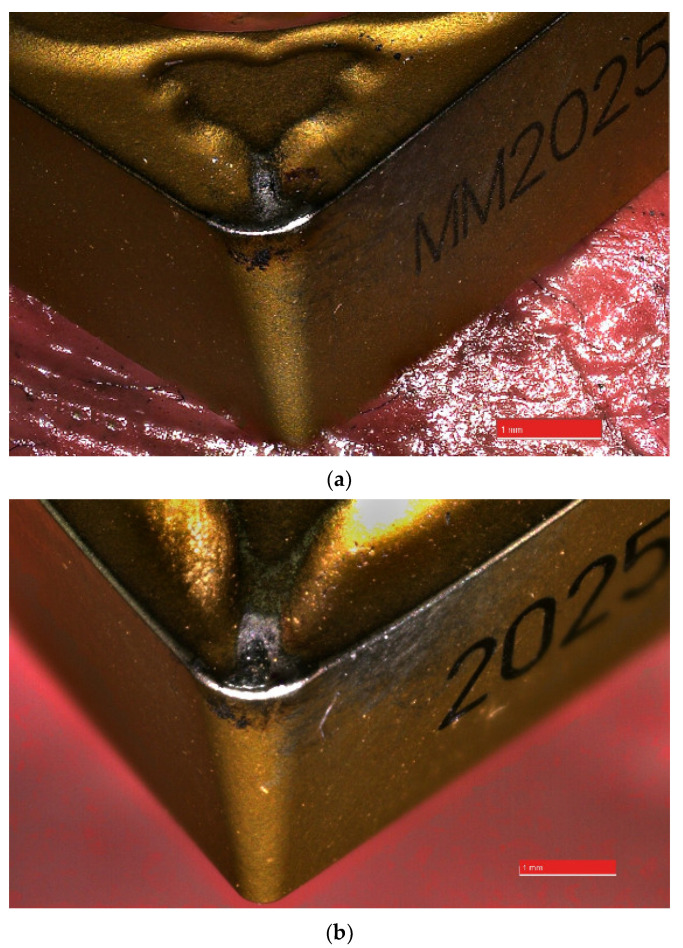
The view of inserts type (**a**) CCMT09T308-MM (CC1); (**b**) CCMT09T308-UM (CC2) after machining with DSS samples.

On Figure 5 the cutting inserts types used to technological process a duplex stainless steel with abrasive wear and build-up was presented.

**Figure 6:**
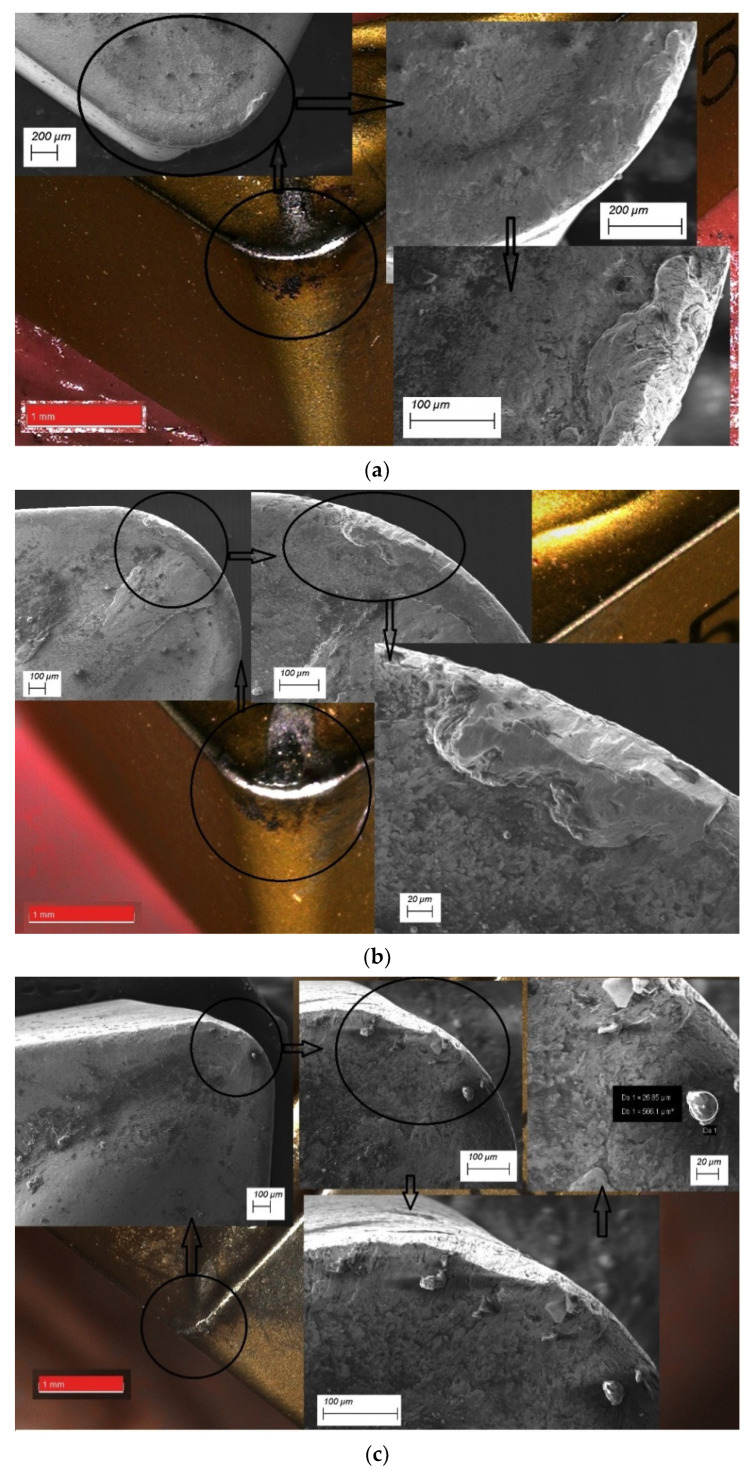
The detailed observation of tool wear for different types of inserts: (**a**) CC1; (**b**) CC2; (**c**) CC3.

Tool wear shown a view on the Figure 6 for the type cutting inserts: CCMT09T308-MM (CC1); CCMT09T308-UM (CC2); CCMT09T304-UM (CC3). These Figures shown views of wear on the rake face and flank. You can see sticks and abrasions at various magnifications and approximations.

**Figure 7:**
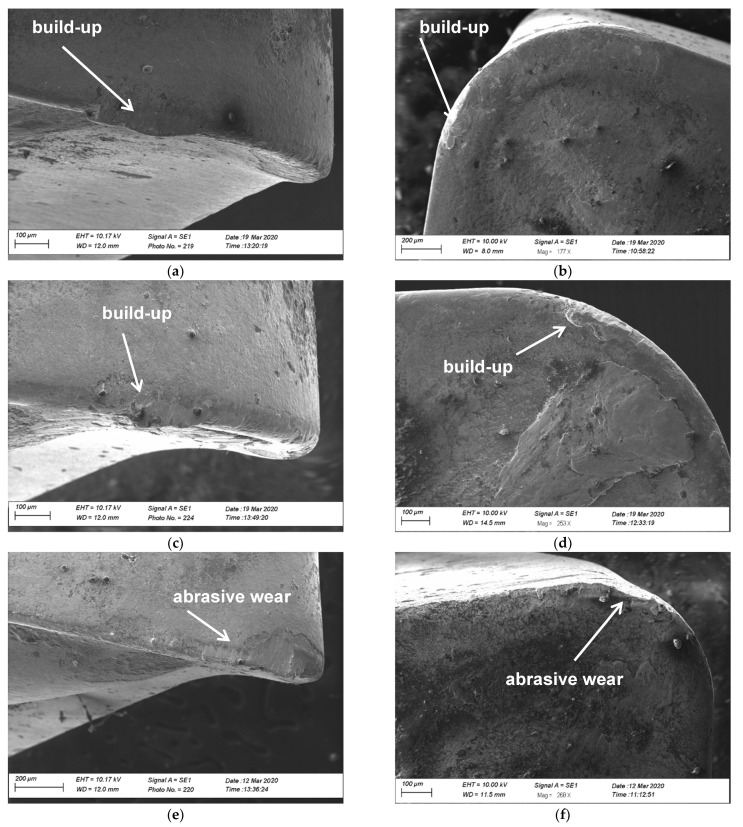
The view of the scanning electron microscopic: (**a**) flank wear for CC1 cutting insert and (**b**) rake face wear for CC1 cutting insert; (**c**) flank wear for CC2 cutting insert and (**d**) rake face wear for CC2 cutting insert; (**e**) flank wear for CC3 cutting insert and (**f**) rake face wear for CC3 cutting insert.

A view of the tool wear of cutting edge from the SEM of the cutting inserts is presented. The state of the used cutting tool assessed surfaces using the adequate images (Figure 7) is presented. The things of wear on the rake face and flank can be observed. For the type and shape cutting inserts for nose radius 0.8 mm, built-up on the flank can be observed. Insert type for a radius of 0.4 mm there is minor tool wear in the form of abrasive wear.

**Figure 8:**
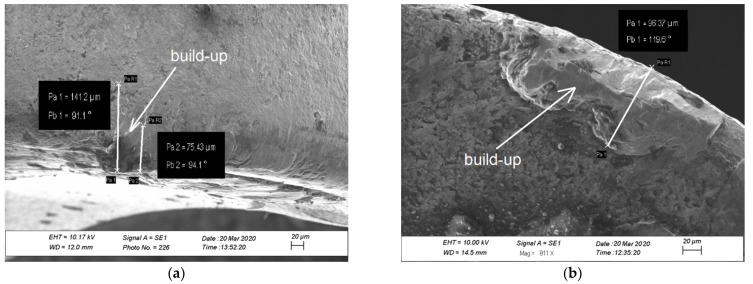
The examples of tool wear measurements by SEM: (**a**) flank wear and (**b**) rake face wear.

In Figure 8 presents examples of measurements of the turning tool wear by SEM. The build-up can be seen on rake face surfaces (Figure 8b). Materials that adhered to the rake face was determined to be DSS. There is very little material adhering to the surface of the tool. It is clearly visible under the magnification of an electron microscope. Therefore, SEM was used for the geometrical measurements of the cutting edge wear.

**Figure 9:**
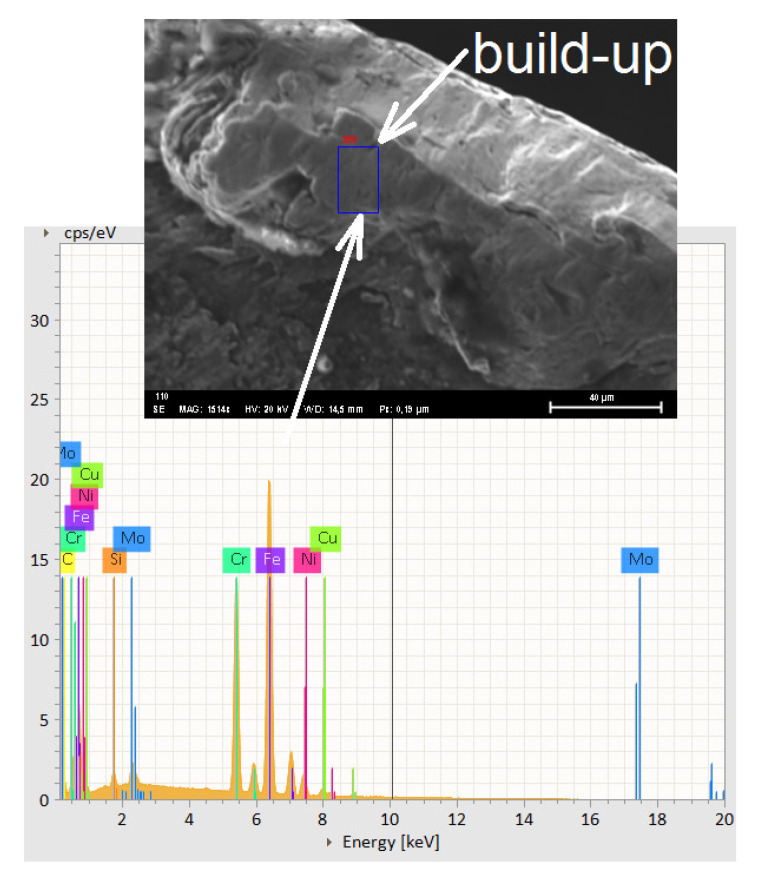
The rake face wear for CC2 cutting insert and EDS analyzer of chemical constitution.

Figure 9 presents the EDS that shows that this is a build-up of DSS. The build-up of DSS determined by the EDS analyzer showing the presence of alloying components characteristic for two-phase stainless steel can be seen. There, build-up, caking and layering from DSS on the race face wear has been attached. It is worth noting that the determination of the chemical constitution (Table 4) with the EDS analyzer is very useful for determining the type of tool wear. You can define whether it is built-up, sticky or abrasion of the tool surface.

Tool wear of flank wear—VB and rake face wear—KB shown a view on the Figure 5, Figure 6, Figure 7 and Figure 8 for the type cutting inserts: CCMT09T308-MM; CCMT09T308-UM; CCMT09T304-UM. Additional materials appear on the cutting inserts through sticking. The effects of the removal of the coating from the cutting insert due to abrasion can also be observed. Careful observation of wear was performed using scanning electron microscopy. The impact of abrasive wear and built-up of the duplex stainless steel material have been observed. Using the EDS analyzer (Figure 9), it was possible to determine chemical constitution of the material adhered to the cutting insert. Therefore, it was found that it was a processed DSS material. The lowest wear was determined for the CC3 cutting insert type. DSS surface for this type of insert was the lowest roughness. The cutting forces were measured using the DKM2010 force meter. During machining, cutting forces can be continuously measured.

As a result, tool wear can also be controlled while cutting. The changes in cutting forces are clearly visible when changing the cutting edge geometry.

## 4. Conclusions

In this paper, tool wear on flank and rake surfaces was presented. The machining duplex stainless steel is difficult. In this work, the appropriate technological parameters choices were made. It is also to obtain the appropriate surface roughness of the DSS.

Tool wear of insert and good surface roughness of surface layer are determined for the given cutting length (*LSC* = 9.75 m).

The turning longitudinal of outside cylindrical surfaces of samples DSS in was carried out by inserts CC09T3 2025 with coating by CVD method.

For samples DSS to get a smooth surface should be carried out using the insert for nose radius *r_ε_* = 0.4 mm and parameters feed rate *f* = 0.1 mm/rev and cutting speed *v_c_* = 70 m/min and depth of cut *a_p_* = 0.5 mm.

Arithmetical mean deviation of the roughness profile after turning was equal *Ra* = 1.05 μm.

Turning of DSS can be processed by insert type CC3, where the low flank wear is VB = 0.036 mm and rake face wear KB = 0.018 mm and surface roughness reduction ratio K*_Ra_* = 3.21 were equal.

Only one insert type CC3 implemented the criterion of quality, where *Ra* parameter is not as much of the value of 1.25 μm and the criterion of wear of the maximum tool wear flank VB = 0.2 mm.

Shaft type machine elements are widely used in mechanical engineering. They can be used in machines such as sea water centrifugal pumps or in petrochemical installations.

The steel DSS in seawater installations for pump shafts is used. Their shaping and finishing is required.

Inserts type CC3 can be used for the technological parameters presented in the paper and for smoothing purposes with low cutting edge wear.

## Figures and Tables

**Table 1:** The chemical constitution and mechanical properties of stainless steel type duplex X2CrNiMoCuN 25–7–4 [%mass].

**C**	**Cr**	**Ni**	**Mo**	**Mn**	**Si**	**N**	**S**	**P**
**[%]**	**[%]**	**[%]**	**[%]**	**[%]**	**[%]**	**[%]**	**[%]**	**[%]**
	24.0	6.0	3.0			0.24		
0.03	26.0	8.0	5.0	1.2	0.8	0.32	0.020	0.035
**R_p0.2_**	**R_m min._**	**R_m max._**	**A_5 min._**	**HV _max._**	**E**	**ρ**	**λ**	**c_w_**
**[MPa]**	**[MPa]**	**[MPa]**	**[%]**	**[-]**	**[GPa]**	**[kg/m^3^]**	**[J/m·s·K]**	**[J/kg·K]**
550	795	1000	15	310	200	7800	14.2	460

**Table 2:** Cutting inserts shape, type and grade for the turning of samples with the X2CrNiMoCuN 25-7-4.

No Cutting Insert	Insert Shape	Insert Type	Insert Grade	Nose Radius [mm]	Flank Angle [°]	Rake Angle [°]
CC1	CC09T3	CCMT09T308-MM	2025	0.8	7	7
CC2		CCMT09T308-UM				6
CC3		CCMT09T304-UM		0.4		

**Table 3:** The parameters of the surface roughness and tool wear of a cutting edge of samples DSS after turning.

No	No	Insert	f	v_c_	VB	KB	Rz	Ra	K_Ra_
Inserts	Samples	Type	[mm/rev]	[m/min]	[mm]	[mm]	[μm]	[μm]	[-]
CC1	M-08-100	CCMT09T308-MM	0.2	100	0.242	0.121	9.21	1.91	1.71
	M-08-70			70	0.194	0.098	8.43	1.65	2.14
	M-08-50			50	0.292	0.147	11.95	2.31	1.43
CC2	U-08-100	CCMT09T308-UM	0.2	100	0.112	0.056	7.58	1.52	2.16
	U-08-70			70	0.097	0.048	7.48	1.55	2.13
	U-08-50			50	0.141	0.071	7.97	1.63	2.02
CC3	U-04-70-2	CCMT09T304-UM	0.2	70	0.064	0.032	13.86	3.03	1.09
	U-04-70-1		0.1	70	0.036	0.018	5.35	1.05	3.21

**Table 4:** The chemical constitution on build-up cutting edge with the EDS analyzer [%mass].

C	Cr	Ni	Mo	Mn	Si	N	S	P
[%]	[%]	[%]	[%]	[%]	[%]	[%]	[%]	[%]
0.03	23.4	5.8	2.8	1.0	0.6	0.2	0.02	0.03

## Nomenclature

SEM	Scanning Electron Microscope
EDS	spectrometer analyzer
DSS	duplex stainless steel
*LSC*	length of spiral cutting, (m)
VB	flank wear, (mm)
KB	rake face wear, (mm)
*D_m_*	diameter of the workpiece, (mm)
*l_m_*	length of the cutting materials, (mm)
*f*	feed rate, (mm/rev)
*a_p_*	depth of cut, (mm)
*v_c_*	cutting speed, (m/min)
